# Poly-Saturated Dolichols from Filamentous Fungi Modulate Activity of Dolichol-Dependent Glycosyltransferase and Physical Properties of Membranes

**DOI:** 10.3390/ijms20123043

**Published:** 2019-06-21

**Authors:** Elżbieta Gryz, Urszula Perlińska-Lenart, Katarzyna Gawarecka, Adam Jozwiak, Sebastian Piłsyk, Agata Lipko, Malgorzata Jemiola-Rzeminska, Przemysław Bernat, Anna Muszewska, Kamil Steczkiewicz, Krzysztof Ginalski, Jerzy Długoński, Kazimierz Strzalka, Ewa Swiezewska, Joanna S. Kruszewska

**Affiliations:** 1 Institute of Biochemistry and Biophysics, Polish Academy of Sciences, Pawinskiego 5a, 02-106 Warsaw, Poland; egryz@ibb.waw.pl (E.G.); lenart@ibb.waw.pl (U.P.-L.); ibb.katag@gmail.com (K.G.); adamj@ibb.waw.pl (A.J.); seba@ibb.waw.pl (S.P.); ag.lipko@ibb.waw.pl (A.L.); musze@ibb.waw.pl (A.M.); ewas@ibb.waw.pl (E.S.); 2 Faculty of Biochemistry, Biophysics and Biotechnology, Jagiellonian University, Gronostajowa 7, 30-387 Krakow, Poland; malgorzata.jemiola@gmail.com (M.J.-R.); kazimierzstrzalka@gmail.com (K.S.); 3 Malopolska Centre of Biotechnology, Jagiellonian University, Gronostajowa 7A, 30-387 Krakow, Poland; 4 Department of Industrial Microbiology and Biotechnology, Faculty of Biology and Environmental Protection, University of Lodz, Banacha 12/16, 90-237 Lodz, Poland; pbernat@biol.uni.lodz.pl (P.B.); jdlugo@biol.uni.lodz.pl (J.D.); 5 Laboratory of Bioinformatics and Systems Biology, CeNT, University of Warsaw, Zwirki i Wigury 93, 02-089 Warsaw, Poland; k.steczkiewicz@cent.uw.edu.pl (K.S.); k.ginalski@cent.uw.edu.pl (K.G.)

**Keywords:** yeast, filamentous fungi, poly-saturated dolichols, dolichyl phosphate mannose synthase, lipidome

## Abstract

Mono-saturated polyprenols (dolichols) have been found in almost all Eukaryotic cells, however, dolichols containing additional saturated bonds at the ω-end, have been identified in *A. fumigatus* and *A. niger*. Here we confirm using an LC-ESI-QTOF-MS analysis, that poly-saturated dolichols are abundant in other filamentous fungi, *Trichoderma reesei*, *A. nidulans* and *Neurospora crassa*, while the yeast *Saccharomyces cerevisiae* only contains the typical mono-saturated dolichols. We also show, using differential scanning calorimetry (DSC) and fluorescence anisotropy of 1,6-diphenyl-l,3,5-hexatriene (DPH) that the structure of dolichols modulates the properties of membranes and affects the functioning of dolichyl diphosphate mannose synthase (DPMS). The activity of this enzyme from *T. reesei* and *S. cerevisiae* was strongly affected by the structure of dolichols. Additionally, the structure of phosphatidylcholine (PC) and phosphatidylethanolamine (PE) model membranes was more strongly disturbed by the poly-saturated dolichols from *Trichoderma* than by the mono-saturated dolichols from yeast. By comparing the lipidome of filamentous fungi with that from *S. cerevisiae*, we revealed significant differences in the PC/PE ratio and fatty acids composition. Filamentous fungi differ from *S. cerevisiae* in the lipid composition of their membranes and the structure of dolichols. The structure of dolichols profoundly affects the functioning of dolichol-dependent enzyme, DPMS.

## 1. Introduction

Polyisoprenoid alcohols are found in all living organisms from bacteria to mammals. They consist of up to more than 100 isoprene residues linked head-to-tail, with a hydroxyl group at one end (α-residue) and a hydrogen atom at the other (ω-end) [1]. In animals and yeast the double bond in the α-residue is hydrogenated, giving dolichols.

In these organisms, dolichols, like all the other isoprenoids, are synthesized via the mevalonate pathway and the first dedicated enzyme of the polyprenol branch of this pathway is *cis*-prenyltransferase (dehydrodolichyl diphosphate synthase) (CPT), a key enzyme in dolichol synthesis. CPT catalyzes the elongation of the polyprenol chain by sequential addition of isopentenyl diphosphate (IPP) to a precursor farnesyl diphosphate (FPP) [2,3,4].

Eukaryotic cells synthesize polyisoprenoids with a specific number of isoprene residues forming a mixture of usually six to eight dolichol species of different chain lengths, while bacteria possess a single polyprenol, undecaprenol (Pren-11) [1]. The pattern of the dolichols varies between kingdoms. Thus, *S. cerevisiae* accumulates a two-family mixture of dolichols—from Dol-14 to Dol-18, with Dol-15 or Dol-16 dominating, and longer ones from Dol-19 to Dol-22, with Dol-21 dominating [5,6,7].

The overall content of dolichols in yeast and *T. reesei* is rather low (approx. 6 µg/g wet weight) [8], but a significant increase has been reported for *S. cerevisiae* at the late logarithmic and early stationary phases (32 and 48 µg/g, respectively) [6]. In contrast, in the human pituitary gland the dolichol content is as high as 7170 µg/g fresh weigh [9].

In addition to the common mono-saturated dolichols, polyisoprenoid alcohols of atypical structures with two or three hydrogenated isoprenoid residues located at the ω-end have been isolated from *Aspergillus fumigatus* [10], and an exo-methylene group was found in tetrahydrodolichols isolated from *A. niger* [11]. These studies showed that also *Trichoderma* synthesizes atypical dolichols not found in other Eukaryotes. While their exact structure has not been reported, it could be expected to have some essential function considering the role played by dolichols.

Dolichols appear to increase the fluidity of phospholipid bilayers or even destabilize lipid membranes, elicit formation of inverted micelles within the membrane, and increase membrane permeability [9]. Dolichyl phosphate (DolP) stimulates vesicle fusion, and in this way plays an important role in protein trafficking between the Golgi, plasma membrane and lysosomes [9,12]. Dolichol and its phosphorylated form are located between the two layers of the phospholipid bilayer, perpendicular to the fatty acyl chains, with the hydrophilic OH- or phosphate group protruding at the outer surface [12,13].

The effect exerted by dolichols on membranes increases with increasing chain length [9]. The length of the polyisoprenoid chain is important for its physico-chemical properties, and this probably relates to the ability to fluidize locally the lipid bilayer [14].

Additionally, the structure of polyisoprenoids has been found to be important for their properties, as exemplified by the different effects on the permeability and fluidity of phospholipid/polyisoprenoid model membranes shown by α-*cis* and α-*trans* isomers [15]. It is therefore reasonable to assume that the activity of the membrane-bound dolichol-dependent enzymes would also be affected—both directly and indirectly—by dolichol composition and structure.

In addition to their effect on membranes, dolichols are important for the cell functioning since dolichyl phosphate is an obligatory carrier of carbohydrate residues during the *O*- and *N*-glycosylation of proteins. In fungi dolichyl phosphate mannose (DPM) delivers the first mannosyl residue to a serine/threonine residue to begin *O*-glycosylation [16]. During *N-*glycosylation, in turn, the whole core oligosaccharide comprised of 14 monosaccharide residues, i.e., tetradecasaccharide, is synthesized on dolichyl phosphate and is then transferred en bloc to a growing polypeptide chain [17,18,19,20]. In addition, *N*-glycosylation requires dolichyl-dependent enzymes also for the synthesis of DPM, the donor of five mannosyl residues, and of dolichyl phosphate glucose (DPG) delivering three glucosyl moieties [20]. A comparison of amino acid sequences of several dolichyl-dependent enzymes has revealed conserved hydrophobic regions of 12–20 amino acids speculated to be the dolichol-binding site [21]. However, site-directed mutagenesis of this region has shown that it is not required for catalytic activity or substrate binding [22]. Whatever the site of its binding, dolichyl phosphate is indispensable for protein glycosylation and its structure could affect this process profoundly.

**Figure 1:**
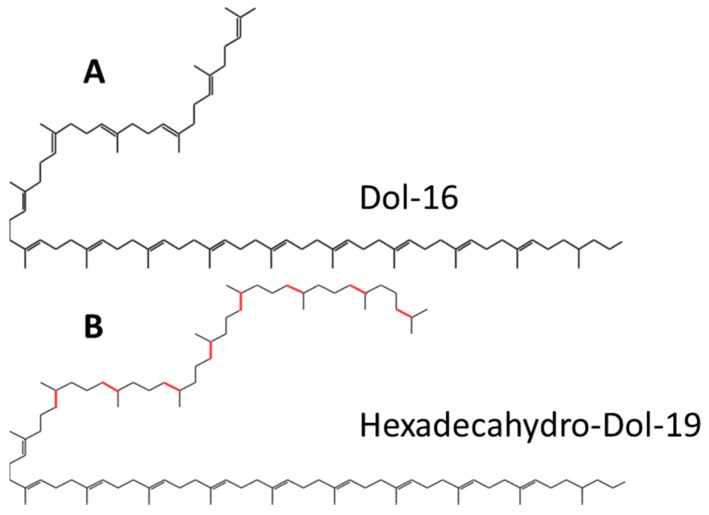
Dominant dolichols in *S. cerevisiae* (**A**) and in *Trichoderma* (**B**). Additional saturated bonds at the omega end in dolichol from *Trichoderma* in red.

In this study we establish the structure and composition of dolichols synthesized by *Trichoderma* and other filamentous fungi. Remarkably, they differ from those of the model Eukaryote and fungus *S. cerevisiae* (Figure 1). We also show how the structure of dolichols affects the activity of dolichyl phosphate mannose (DPM) synthase (DPMS). 3D models of DPMS from *S. cerevisiae* and *T. reesei* in relation to the respective dolichol structure are used to predict modes of dolichol processing. In addition, the influence of the dolichols of different structure on the physico-chemical properties of model phospholipid membranes is established.

## 2. Results

### 2.1. Structure of Dolichols from Trichoderma reesei

An initial HPLC/UV analysis of polyprenols isolated from *T. reesei* revealed a complex mixture containing numerous additional compounds besides those found in mammalian or *S. cerevisiae* cells and eluting with retention times corresponding to Dol-18–Dol-20. This was confirmed by the finer liquid chromatography electrospray ionization-quadrupole-time of flight-mass spectrometry (LC-ESI-QTOF-MS, liquide chromatography electrospray ionization-quadrupole-time of flight-mass spectrometry) analysis again showing a much more complex mixture of compounds (Table 1, Figure S1) than that reported for *S. cerevisiae* (Dol-14 to Dol-18 with Dol-16 dominating) [5,6,7]. An LC-ESI-QTOF-MS analysis showed that the polyprenols from *T. reesei* comprise a complex mixture of compounds with 18, 19 or 20 isoprene units and an apparently different degree of saturation (Table 1, a full list is presented in Table S1). In addition to the typical α-saturated dolichols, i.e., Dol-18, -19 and -20, poly-saturated molecules with between two and ten saturated bonds were observed. Altogether, 31 species could be identified. Basing on the reported structure of the atypical dolichols found in *Aspergillus fumigatus* [10] and the hexa-hydro-polyprenols, glycinoprenols from leaves of several plant species [23], in which the additional saturated bonds are located at the ω-end of the molecules, we postulate that also the *T. reesei* polyprenols have such a structure, i.e., that they contain a single saturated α-residue and varying numbers (1 to 10) of saturated residues stretching from ω-terminal one (Figure 1).

To determine whether such poly-saturated structure is typical for dolichols from filamentous fungi, we analyzed dolichols from two more species, *A. niger* and *N. crassa*. Indeed, additional saturated bonds were found in the Dols isolated from both species (Table 1), albeit the degree of saturation only one or two extra bonds—was far less than that found for *T. reesei*. Both species also contained standard mono-saturated dolichols. Interestingly, longer species than those in *T. reesei* were detected (up to Dol-23 in *A. niger* and up to Dol-20 in *N. crassa*). One can thus conclude that polysaturated dolichols are in fact typical for filamentous fungi and that their length distribution and degree of saturation differ markedly between species.

### 2.2. Dolichol Content in S. cerevisiae and Filamentous Fungi

We reasoned that the markedly different composition and structure of dolichols from filamentous fungi and yeast could affect their overall content in the cell. To verify this assumption dolichols as a class were quantified in four fungal species.

**Figure 2:**
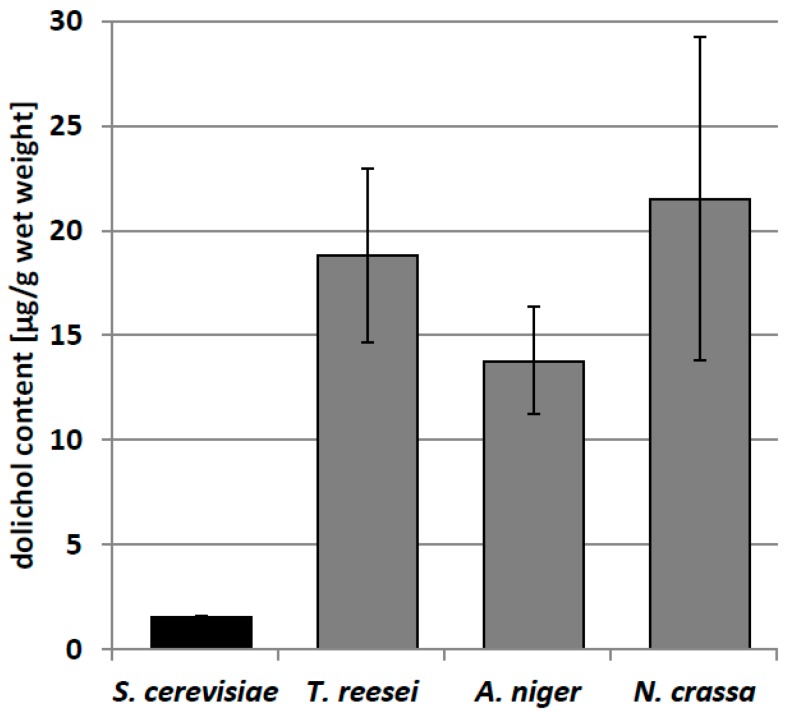
Dolichol content in yeast and filamentous fungi. Data are mean ± standard deviation from three independent experiments.

The content of dolichols was similar in the three filamentous fungi studied, but was markedly (8–10 times) higher than in yeast (Figure 2).

### 2.3. Activity of Dolichyl Phosphate Mannose Synthase

To study how the unusual structure of the *T. reesei* dolichols affects the activity of dolichol-dependent enzyme DPMS, we used various dolichyl phosphates as an acceptor of mannosyl residue.

Native dolichols from *Trichoderma* and *S. cerevisiae* were chemically phosphorylated and then used as substrates in reactions catalyzed by dolichol-dependent ER membrane-bound glycosyltransferase, dolichyl phosphate mannose synthase (DPMS).

Membrane fractions isolated from *T. reesei* and *S. cerevisiae* were used as a source of this enzyme, and the phosphorylated dolichols from *Trichoderma* and yeast were added as exogenous acceptors to the reaction mixture. In parallel, the same reaction was carried out with phosphorylated hepatic dolichols (Dol-18/19-P) and phosphorylated semisynthetic analogue of Dol-11, α-dihydro-Prenol-11 (dHPren11-P, Collection of Polyprenols, IBB PAS). The activity of DPMS was clearly affected by the type of Dol-P used in the assay. DPMS from *S. cerevisiae* showed the highest activity with its cognate substrate ScDol-P, albeit a very high amount of ScDol-P (44 µg) was needed to obtain the maximal efficiency (Table 2). The activity was lower with dHP11-P and with the phosphorylated hepatic dolichols, but the affinity for these dolichols was markedly higher (as reflected by the lower values of *K*_m_, see Table 2). Most importantly, the phosphorylated poly-saturated dolichols from *Trichoderma* (TrDol-P) were by far the poorest acceptors of mannosyl residues for the yeast enzyme. Its maximum activity was 7-fold lower than with the yeast dolichols and reaching this activity required 73 µg of TrDol-P. Accordingly, the *K*_m_ value was nearly double that for the cognate acceptor (Table 2).

The activity of DPMS from *T. reesei* (TrDPMS) was, in general, much lower than that of the yeast enzyme for all analyzed dolichols (Table 2). The highest activity was obtained using the mono-saturated dolichols from *S. cerevisiae*, although their amount required for maximal activity was as high as 73 µg. TrDPMS had the highest affinity for the hepatic dolichols as did the yeast enzyme, and their *K*_m_ values were nearly identical (Table 2). Perhaps unexpectedly, the poly-saturated dolichyl phosphates from *T. reesei* turned out to be the poorest substrate also for the *T. reesei* DPMS.

### 2.4. Modeling of DPMS Structure

To determine the structural features of the DPMS from yeast and *T. reesei* responsible for their different affinities for specific dolichol phosphates, we resorted to homology modelling. We were particularly interested in explaining the reason behind the poor use of the poly-saturated dolichols by both enzymes, since the additional saturated bonds lie far away from the phosphoester end of the molecule serving as the acceptor of the mannosyl moiety.

**Figure 3:**
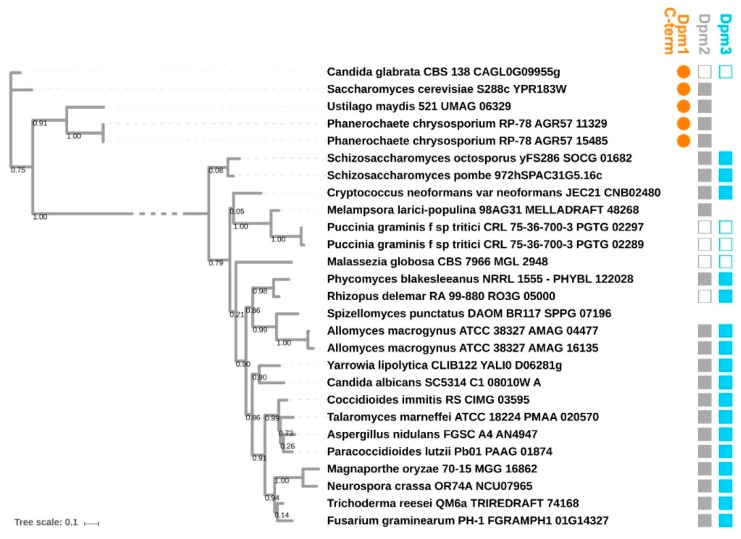
Maximum likelihood phylogenetic tree of catalytic subunit Dpm1 of dolichyl diphosphate mannose synthase (DPMS) in selected representative fungi. Species names are followed by gene identifier. Orange circles denote presence of C-terminal hydrophobic α-helix in Dpm1, grey and blue squares indicate presence of Dpm2 and Dpm3, respectively. Empty squares indicate that the corresponding subunit has not been reported for a given species but is present in other species from this genus. Bootstrap support values are provided at corresponding branches.

DPMSs are monomeric or form heterooligomers of up to three subunits Dpm1, Dpm2 and Dpm3. Dpm1 is the catalytic subunit belonging to Glycos_transf_2 family (PF00535) of glycosyltransferases, widely conserved across all kingdoms of life. Dpm2 (PF07297) is referred to as a regulatory subunit, while Dpm3 (PF08285) tethers the complex at the reticulum membrane [24]. Fungal DPMSs can be divided into two major phylogenetic clades based on their Dpm1 subunit (Figure 3): Type I (including DPMS from *S. cerevisiae*) and II (including DPMS from *Trichoderma*), as discussed in [24,25,26,27,28]. Type I DPMSs consist exclusively of the Dpm1 subunit, which uses its additional C-terminal α-helix for tethering to the lipid bilayer. Type II DPMSs contain the additional Dpm3 subunit anchoring Dpm1 at the membrane.

**Figure 4:**
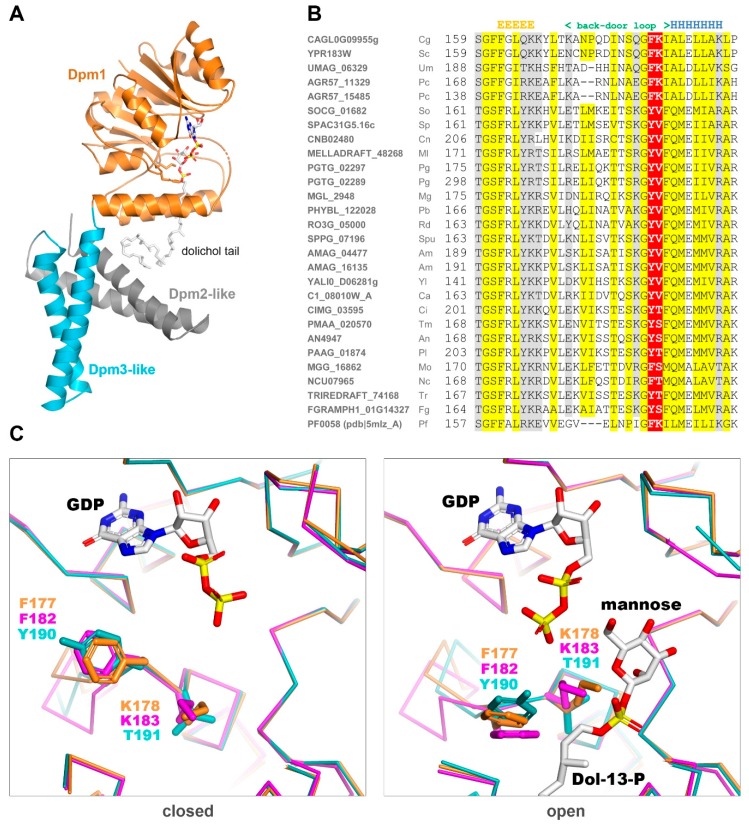
3D model of DPMS. (**A**) Overall structure of *P. furiosus* DPMS in complex with guanosine diphosphate (GDP) and dolichyl phosphate mannose (dHPren-11-P) (pdb|5mm1) (**B**) Multiple sequence alignment of the “back door” loop region of Dpm1. Residue conservation is denoted with the following scheme: hydrophobic positions—highlighted in yellow, polar amino acids—highlighted in grey. The FK motif or its equivalent is highlighted in red. Species names are abbreviated as follows: Cg, *Candida glabrata* CBS 138; Sc, *Saccharomyces cerevisiae* S288c; Um, *Ustilago maydis* 521; Pc, *Phanerochaete chrysosporium* RP-78; So, *Schizosaccharomyces octosporus* yFS286; Sp, *Schizosaccharomyces pombe* 972h-; Cn, *Cryptococcus neoformans* var. neoformans JEC21; Ml, *Melampsora larici-populina* 98AG31; Pg, *Puccinia graminis* f. sp. *tritici* CRL 75-36-700-3; Mg, *Malassezia globosa* CBS 7966; Pb, *Phycomyces blakesleeanus* NRRL 1555; Rd, *Rhizopus delemar* RA 99-880; Spu, *Spizellomyces punctatus* DAOM BR117; Am, *Allomyces macrogynus* ATCC 38327; Yl, *Yarrowia lipolytica* CLIB122; Ca, *Candida albicans* SC5314; Ci, *Coccidioides immitis* RS; Tm, *Talaromyces marneffei* ATCC 18224; An, *Aspergillus nidulans* FGSC A4; Pl, *Paracoccidioides lutzii* Pb01; Mo, *Magnaporthe oryzae* 70-15; Nc, *Neurospora crassa* OR74A; Tr, *Trichoderma reesei* QM6a; Fg, *Fusarium graminearum* PH-1; Pf, *Pyrococcus furiosus*. (**C**) 3D models of active centre region of Dpm1 in closed and open form from *S. cerevisiae* and *T. reesei* superimposed onto PfDPMS structure in “back door” closed (pdb|5mlz) and open (pdb|5mm1) states. *P. furiosus* structure is shown in orange while *Trichoderma* and yeast models in teal and magenta, respectively.

The only DPMS with a known 3D structure is the one from *Pyrococcus furiosus*, PfDPMS, whose structure in complex with dHPren-11-P has been solved recently [28] (Figure 4A).

This enzyme has an architecture different from both Type I and II DPMSs since its single subunit carries two additional transmembrane subdomains at the C-terminus. These additional fragments lack detectable sequence similarity to Dpm2 or Dpm3 from fungi or any other Eukaryote. Yet, PfDPMS is still highly similar in sequence to the fungal Dpm1s (42% and 37% amino acid sequence identity to Dpm1 from *S. cerevisiae* and *T. reesei*, respectively). Structural study on PfDPMS has provided invaluable information about the dolichol binding mode. Based on the *P. furiosus* PfDPMS structures solved in two consecutive states (closed: pdb|5mlz and open: pdb|5mm1), we built 3D models for the catalytic domains of Dpm1from *S. cerevisiae* and *T. reesei* in the open and closed “back door” states. Regarding the catalytic cavity outline, the two fungal Dpm1 clades differ substantially in the “back door” loop sequence. While yeast Dpm1 carries the ^182^FK motif present in *P. furiosus*, the majority of fungi have a YT (tyrosine, threonine) motif instead (Figure 4B). The lysine residue following phenylalanine is important for dolichol stabilization in the active site and its lack suggests weaker substrate binding.

### 2.5. Phospholipid Profile of Membranes from Filamentous Fungi and S. cerevisiae

The structure of *P. furiosus* DPMS shows that the dolichol chain is buried in the lipid bilayer and interacts mainly with the transmembrane subdomains of DPMS. This result pointed at the importance of the membrane environment, determined chiefly by phospholipids, for the DPMS–dolichol interaction. Ergosterol could also be predicted to be a good test lipid to emphasize the difference between dolichols from different species having different amounts of saturated bonds. Its amount in dry mass of *S. cerevisiae* and *Trichoderma* was 5.43 ± 0.23 and 4.23 ± 0.29 mg/g dry mass respectively.

Next, we compared the phospholipid profiles of cellular membranes from filamentous fungi and *S. cerevisiae*. In addition to highlighting differences between these two groups of fungi possibly affecting the functioning of their DPMSs, the study also established which phospholipids are dominant in the membranes of *T. reesei* and S*. cerevisiae*. This data allowed us to choose proper model membranes for further biophysical studies on the influence of dolichol structure on the thermotropic properties of the model membranes.

**Figure 5:**
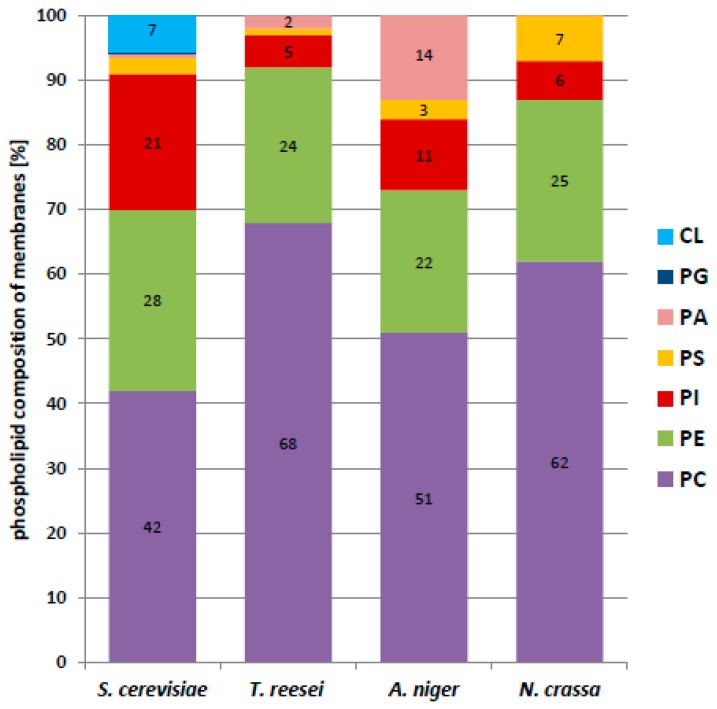
Phospholipid class composition of membranes from yeast and filamentous fungi. PA—phosphatidic acid; PC—phosphatidylcholine; PE—phosphatidylethanolamine; PG—phosphatidylglycerol; PI—phosphatidylinositol; PS—phosphatidylserine; CL—cardiolipin.

We found that phosphatidylcholine (PC) was the dominant phospholipid in all the cells examined (Figure 5). In filamentous fungi its content in the membrane fraction varied from 68% in *T. reesei* to 51% in *A. niger*, and it was much less abundant, albeit still dominant in *S. cerevisiae*. In contrast, phosphatidylethanolamine (PE) constituted a similar fraction of phospholipids in all the fungi, from 27.6% in *S. cerevisiae* to 21.6%. in *A. niger*. The PC/PE ratio plays a key role in membrane integrity and in the maintenance of its function. PC has stabilizing properties whereas PE forms non-bilayer hexagonal phases [29,30]. In the present study, the PC/PE ratio was fairly high and quite similar for the filamentous fungi, 2.4 for *A. niger*, 2.5 for *N. crassa*, and 2.8 for *T. reesei*, but only 1.5 for *S. cerevisiae*.

The yeast membranes differed from the others also in the much higher content of phosphatidylinositol (PI) (20.6% vs. 4.9–11.5%). Notably, yeast membranes contained cardiolipin (CL) at a fairly high proportion (6.6%), which was not found in the filamentous fungi.

**Figure 6:**
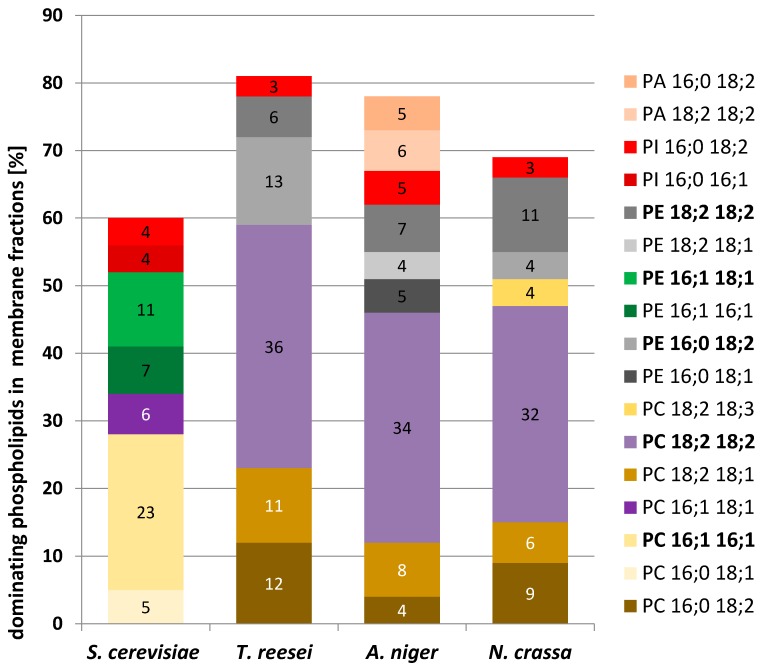
Most abundant phospholipid species in membrane fractions from yeast and filamentous fungi. PA—phosphatidic acid; PC—phosphatidylcholine; PE—phosphatidylethanolamine; PI—phosphatidylinositol; 16:0 palmitic acid; 16:1 palmitoleic acid; 18:1 oleic acid; 18:2 linoleic acid; 18:3 γ-linolenic acid.

A more detailed mass spectrometry analysis of the membrane phospholipids showed further differences in the pattern of their fatty acids (Table 3, Figure 6). While in filamentous fungi the most abundant phospholipid species were those containing mostly linoleic acid (C18:2), palmitoleic acid (C16:1) dominated in *S. cerevisiae*.

### 2.6. Influence of Dolichols of Different Structure on Physico-Chemical Properties of Model Phospholipid Membranes

The local environment of the membrane-bound dolichol-dependent enzymes is likely to affect their functioning. In turn, the presence of dolichol in the lipid bilayer will modulate the membrane properties and hence, indirectly, also the properties of the relevant enzymes. These effects could depend on both the composition of the membrane and the structure of the dolichols.

We therefore set out to check if mono- and poly- saturated dolichols (i.e., ScDol and TrDol, respectively) could differently alter the properties of model membranes of different composition.

Since phosphatidylcholine (PC) and phosphatidylethanolamine (PE) were found to dominate in membranes from filamentous fungi and *S. cerevisiae* (Figure 5), we decided to use two types of model membranes composed exclusively of a single species of PC or PE, 1,2-dipalmitoyl-*sn*-glycero-3-phosphatidylocholine (DPPC) or 1,2-dimyristoyl-*sn*-glycero-3-phosphatidylethanolamine (DMPE), respectively. Using chemically pure phospholipids rather than a mixture of molecular species gave more defined data and simplified their interpretation. Those phospholipids supplemented with different proportion of Tr or Sc dolichols were used to obtain multilamellar vesicles (MV) and their thermal stability was determined. Although ergosterol is present in fungal membranes in a significant amount and is known to affect membrane fluidity we used DPPC and DMPE model membranes supplemented with dolichols not to introduce the third player and make the experiment simpler. 

**Figure 7:**
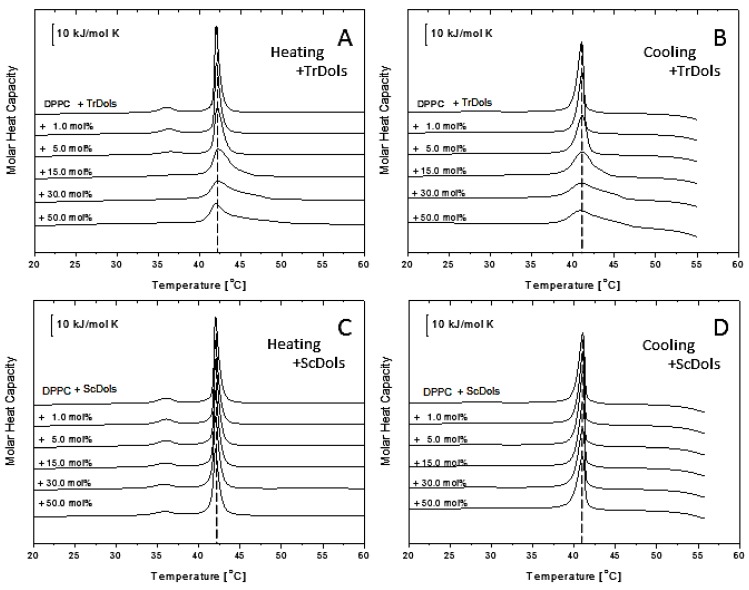
Influence of dolichols on thermal properties of 1,2-dipalmitoyl-*sn*-glycero-3-phosphatidylocholine (DPPC) membranes. Differential scanning calorimetry (DSC) of multilamellar vesicles (MV) obtained in the heating (**A**,**C**) and cooling (**B**,**D**) modes for TrDols (**A**,**B**) and ScDols (**C**,**D**) are shown. Scans were obtained at the rate of 1 °C min^−1^; representative scans of three independent runs are shown.

Figure 7 shows thermal curves for differential scanning calorimetry (DSC) experiments for DPPC multilamellar vesicles upon heating (A and C) and cooling (B and D). Upon heating, fully hydrated DPPC showed an endothermic peak with the main transition temperature of 42.02 ± 0.1 °C and an enthalpy change (ΔH) of 31.69 kJ.mol^−1^ (Table 4), corresponding to the conversion of the gel phase (L_β’_) to liquid-crystalline phase (L_α’_). A smaller transition from the lamellar gel phase (L_β’_) to the rippled gel phase (P_β’_), called a pretransition, centered at 14.91 °C with ΔH of 6.72 kJ.mol^−1^ (Table 4) was recorded. These values for the pure DPPC system are in good agreement with those reported earlier [31,32].

**Figure 8:**
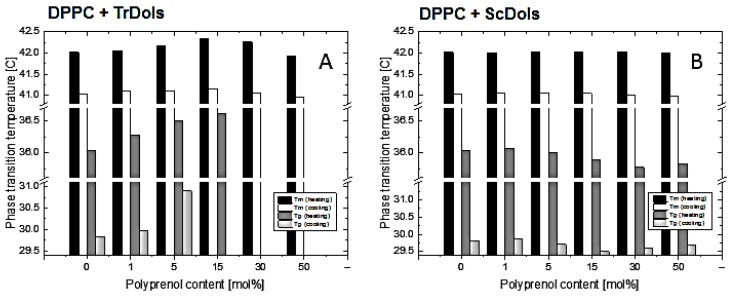
Influence of concentration of dolichol on phase transition temperature of DPPC membranes. Phase transition temperature of DPPC multilamellar vesicles obtained in the cooling and heating modes for TrDols (**A**) and ScDols (**B**).

As shown in Figure 7A,B, dolichols isolated from *Trichoderma*, TrDol, significantly altered the thermotropic phase behavior of the MVs. The height of the main melting peak decreases progressively with an increasing dose of TrDol, which was accompanied by its progressive broadening. Such changes indicate that dolichols strongly decrease the cooperativity of the main phase transition, while the enthalpy changes by no more than 7% (Table 4). Moreover, the peak becomes asymmetric, especially when the dolichol/DPPC molar ratio exceeds 15 mol%. In agreement with the small ∆H change, the T_m_ is weakly affected: it increases slightly up to 15 mol% and then drops below the T_m_ of pure DPPC for 50 mol% (Table 4, Figure 8).

Concurrently with the disturbance of the gel phase-to-liquid-crystalline phase transition, TrDols also modulate the pretransition, which becomes progressively smaller, shifted to higher temperatures and finally disappears at 30 mol% upon heating (Figure 7A). This latter effect is even more apparent during cooling, as the pretransition peak is hardly visible already at 5 mol%.

In contrast to the dolichols from *Trichoderma*, yeast dolichols, ScDol, had almost no effect on the DPPC thermotropic phase behavior as evidenced by the virtually unaffected heating and cooling traces shown in Figure 7C,D. Furthermore, T_m_ did not depend on the dolichol content, and only a slight decrease of T_p_, not higher than 0.32 °C, was found.

**Figure 9:**
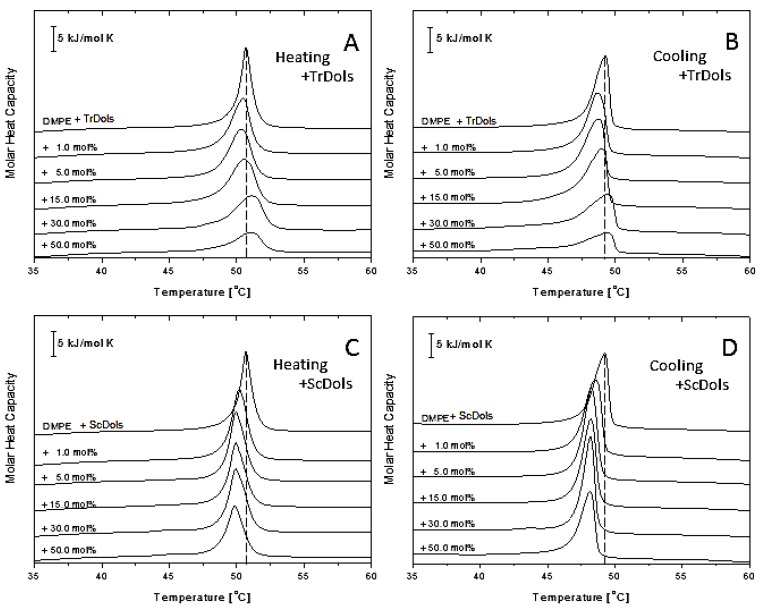
Influence of dolichols on thermal properties of 1,2-dimyristoyl-*sn*-glycero-3-phosphatidylethanolamine (DMPE) membranes. Differential scanning calorimetry (DSC) of multilamellar vesicles obtained in the heating (**A**,**C**) and cooling (**B**,**D**) modes for TrDols (**A**,**B**) and ScDols (**C**,**D**) are shown. Scans were obtained at the rate of 1 °C min^−1^: representative scans of three independent runs are shown.

Figure 8 shows similar thermograms obtained for DMPE MVs. As reported previously [33], DMPE in a well hydrated state presents a single and strong transition peak corresponding to the gel-to-liquid-crystalline phase transition. In our DSC experiments it was found at 50.67 °C upon heating, with a transition enthalpy of 22.84 kJ·mol^−1^ (Table 5). Upon cooling (Figure 9B,D), the peak becomes shifted by 1.41 °C and broadens markedly.

**Figure 10:**
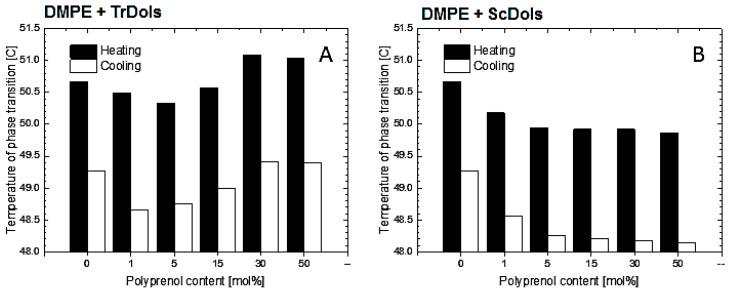
Influence of concentration of dolichol on phase transition temperature of DMPE membranes. Phase transition temperature of DMPE multilamellar vesicles obtained in the cooling and heating modes for TrDols (**A**) and ScDols (**B**).

Incorporation of dolichols isolated from *Trichoderma* or from yeast markedly altered the thermotropic behavior of DMPE, albeit in a strikingly different manner. In the case of *Trichoderma* dolichols, lowering and broadening of the phospholipid transition peak is observed (Figure 9A,B), which again reflects a reduced cooperativity between the PE acyl chain. Additionally, T_m_ decreases by 0.34 °C for 5 mol% and then increases at higher dolichol/PE molar ratios with saturation at 50 mol% (Table 5, Figure 10A). The same feature was observed upon cooling.

Unlike the *Trichoderma* dolichols, ScDols failed to modify the shape of the DMPE phase transition curve (Figure 9C,D). However, as show in Figure 10B, they did destabilize the DMPE, as the T_m_ was lowered significantly at such a low dolichol content as 1 and 5 mol% and remained low at the higher dolichol/ DMPE molar ratios.

**Figure 11:**
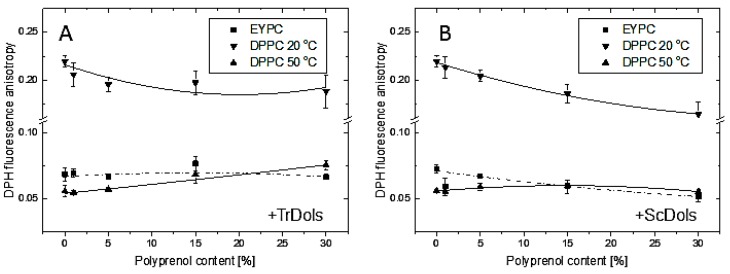
Influence of concentration of dolichol on fluorescence anisotropy of l,6-diphenyl-l,3,5-hexatriene (DPH) in egg yolk lecithin (EYPC) and 1,2-dipalmitoyl-*sn*-glycero-3-phosphatidylocholine (DPPC) liposomes. DPH fluorescence anisotropy in EYPC and DPPC multilamellar vesicles containing different amount of TrDols (**A**) and ScDols (**B**) Continuous lines between points represent theoretical fits to the experimental data by applying equation: y = Intercept + B1 × x^1^ + B2 × x^2^ (Table S2).

To elucidate the influence of the dolichols on the phospholipid bilayer order, we performed fluorescence anisotropy studies. The dependence of the anisotropy of fluorescence of l,6-diphenyl-1, 3,5-hexatriene (DPH) on the content of dolichols from *Trichoderma* and yeast in DPPC liposomes at 20 °C and 50 °C (i.e., below and above the phase transition temperature, respectively) as well as in small unilamellar vesicles (SUVs) formed from egg yolk lecithin (EYPC) at 20 °C (liquid crystalline phase), is shown in Figure 11.

Dolichols, especially those from yeast, fluidize the highly ordered DPPC bilayer at 20 °C, when it is in the gel state. Conversely, they exert a stabilizing effect at 50 °C, i.e., on the liquid-crystalline state, and in this case the *Trichoderma* dolichols (Figure 11A) are more effective. Both these effects are concentration-dependent. On the other hand, the egg yolk lecithin (EYPC) bilayer studied above its phase transition temperature was virtually unaffected by TrDols and slightly destabilized by ScDols.

Thus, our studies showed that the effect of dolichols on the physicochemical properties of model phospholipid membranes depends on the dolichol structure and concentration and also on the phospholipid class.

### 2.7. Activity of DPMS from Trichoderma Expressed in S. cerevisiae

Our studies show that *Trichoderma* and *S. cerevisiae* differ in structure of dolichols and concentration and class of phospholipids in their membranes.

The overlap of the two factors that may affect the activity of dolichol-dependent membrane-bound enzymes such as DPMS makes it difficult to draw conclusions about the role of dolichol in the regulation of activity of this enzyme. To avoid the problem, we analyzed the activity of DPMS from *Trichoderma* expressed in *S. cerevisiae*. For this study, we chose an *S. cerevisiae* mutant carrying the Dpm1 and Dpm3 proteins from *Trichoderma*. The Dpm2 regulatory protein was omitted since this way DPMS from *Trichoderma* is regulated only by the factors present in yeast [25,27].

The activity of DPMS from *Trichoderma* (TrDPMS-1,3) located in the yeast membranes was in general, similar to the activity of the native yeast enzyme (ScDPMS) (Table 2). Both enzymes, the *Trichoderma* (TrDPMS-1,3) and the yeast one, were affected by the type of Dol-P the same way. The highest activity was observed with ScDol-P and the lowest with TrDol-P as substrates.

Affinity of TrDPMS-1,3 for phosphorylated poly-saturated dolichols from *Trichoderma* (TrDol-P) was markedly lower (as reflected by the higher values of *K*_m_, see Table 2) compared to the hepatic dolichols or dHP11-P.

These results clearly confirm the crucial role of the structure of dolichols in the regulation of DPMS activity.

## 3. Discussion

The only dolichols from filamentous fungi described so far were those isolated from *A. niger* [11] and *A. fumigatus* [10], and rather unexpectedly they were found to differ structurally from typical dolichols, as exemplified by those from animals or *S. cerevisiae*. In addition to these structural differences, they were also longer than the yeast ones, which are up to 22 isoprene units long [5,6,7]. *A. niger* contained a family of exo-methylenehexahydroprenols of between 18 and 24 isoprene residues, and *A. fumigatus* had dolichols with two or three hydrogenated isoprenoid residues located at the ω-end.

Our studies show that dolichols from all three species of filamentous fungi examined, representing three evolutionarily distant genera, contain additional saturated bonds. We believe that one can assume that this “unusual” structure is in fact typical for dolichols from filamentous fungi—until exceptions are found.

This observation raises the question of why these dolichols differ from those found in other Eukaryotes and what are the consequences of their presence in the cell.

To answer these questions, we compared the influence of the typical mono-saturated dolichols vs. the poly-saturated ones on the activity of dolichol-dependent enzyme and on the properties of model lipid membranes.

The enzymatic activity of DPMS was strongly dependent on the structure of the dolichols used as the carbohydrate acceptor. The *Trichoderma* enzyme showed relatively low activity with the cognate TrDol-P, while the yeast enzyme, although highly active with the native yeast ScDol-P, required an extremely high concentration thereof for maximal activity. These results show that not only the dolichol structure but also its concentration critically affect the activity of the enzyme. We found that the concentration of dolichols in *Trichoderma* and yeast is not as high as in human organs, where the liver contains 452 µg of dolichols per g of wet weight and the pituitary gland, the organ richest in dolichols, up to 7170 µg/g w.w. [9]. It has been reported that only a small and variable fraction of dolichols is phosphorylated: as a consequence, the amount of Dol-P, which is the only form active as the acceptor of carbohydrates, is not proportional to the overall Dol content in a given organ [9,34].

The DPMS from *S. cerevisiae* was much more active than that from *Trichoderma*. At a given concentration of native Dol-P the yeast DPMS exhibited from 8- to 55-fold higher activity. The dolichyl phosphate mannose formed by DPMS is mostly used for protein glycosylation, GPI-anchor formation and the synthesis of the cell wall [35,36,37]. Thus, one could expect a positive correlation between the requirement for dolichyl phosphate mannose for these processes and the speed for its synthesis. Indeed, the content of mannose in the cell wall is substantially higher for *S. cerevisiae* (279 µg of mannose /mg dry cell wall, Kruszewska, unpublished) than for *Trichoderma* (127 µg of mannose /mg dry cell wall) [38]. In addition to the intrinsic differences in the activity of DPMS from these two species it could be additionally modulated by the structure of their cognate Dol-P substrates. The mechanism of the latter effect is, however, difficult to fathom.

The additional saturation of the fungal dolichols most likely concerns their ω-end which is distant from the active centre. The substrate specificity of DPMS has been shown to be high towards the sugar moiety (GDP-Man strongly preferred) but rather weak towards the Dol-P acceptor. The latter is justified by the structure of the DPMS from *P. furiosus*, which demonstrates that the dolichol chain is buried within the lipid bilayer and interacts mainly with the transmembrane subdomains of DPMS. Unfortunately, due to the lack of detectable homology between the *P. furiosus* DPMS transmembrane domains and the fungal Dpm2 and Dpm3 subunits we were unable to model the quaternary structure of the fungal DPMS complex, and consequently its interaction with dolichols of different structure. The differences in the composition of the transmembrane domains between the two major DPMS types, as well as between DPMSs and other glycosyltransferases also belonging to the Glycos_transf_2 family (e.g., GtrB tetramerizes through its two transmembrane α-helices, pdb|5ekp [39]) suggest the possibility of profound differences in their quaternary structures.

The observed differences in the kinetic parameters of the two DPMSs could also be a consequence of minor alterations in the catalytic subunit itself. As proposed by Gandini et al. [28], the mannosylation reaction catalyzed by DPMS takes place in a shielded active site pocket, which cannot be vacated for the next reaction round by simple diffusion. Instead, conformational shifts are needed to open the reaction site to allow the replacement of GDP with a new molecule of GDP-mannose and then close it—all potentially dependent on the physicochemical properties of the closing loop. The differences in the amino acid sequences of this loop between *Trichoderma* and *S. cerevisiae* suggest that the yeast enzyme, similarly to that from *Pyrococcus*, would better stabilize the bound Dol-P through an ionic interaction between lysine (K183) and its phosphate moiety, as well as keep the “back door” more securely closed with the large hydrophobic side group of phenylalanine (F182). In most fungi, including *Trichoderma* and *Neurospora*, DPMS lacks the lysine from the “FK” motif, which suggests that the interaction with dolichol phosphate is rather weak.

Additionally, the ultimate significance of Dpm2 and Dpm3 subunits and the transmembrane helices of Dpm1 is unknown. The *S. cerevisiae* Dpm1 can complement human and *Schizosaccharomyces pombe* cells deficient in DPMS activity [26] but not vice versa, which is consistent with the presence of a membrane-tethering element in the former. However, *Pyrococcus* DPMS remains functional even when all its C-terminal transmembrane helices are trimmed off, which additionally complicates the picture of the interaction between dolichol and DPMS [28].

Likewise, site-directed mutagenesis of the putative dolichol binding sequence at the C-terminus of *S. cerevisiae* DPMS indicated that the amino acid sequence of the conserved domain was not critically important for Dol-P binding when the enzyme was embedded in a lipid matrix [22].

On the other hand, studies by Zhou and Troy [13] on peptides containing polyisoprenoid recognition sequence (PIRS) identified in Dpm1p and Alg7p from yeast confirmed the docking of Dol-P to PIRS and alteration of the conformation and motional properties of both partners in the complex altered by the phospholipid surrounding of Dol-P and DPMS in the membrane. Zhou and Troy [13] have reported that incorporation of dolichol or Dol-P into membranes disturbs the phospholipid bilayer in the surrounding region. It is reasonable to expect that a reciprocal influence would also take place, i.e., that the properties of the lipid bilayer would affect the behavior of dolichol and hence also its interaction with a membrane-bound enzyme. Moreover, this effect should depend on the dolichol structure (chain length and the presence or not of extra saturated bonds). Thus, the different phospholipid composition of membranes in yeast and *Trichoderma* combined with the different structure of their dolichols could affect the functioning of dolichol-dependent enzymes in a markedly different manner. However, when comparing the activity of DPMS from yeast (ScDPMS) and *Trichoderma* (TrDPMS1,3) located in the same yeast membranes, it can be concluded that the structure of dolichols plays a key role in regulating their activity.

It has been reported that PC is the most abundant phospholipid in *S. cerevisiae* and *A. niger* [40,41]. Our analysis of membranes from *S. cerevisiae* and filamentous fungi showed the dominance of PC and PE in all species albeit the PC/PE ratio was much higher in filamentous fungi than in yeast. Since PC stabilizes the phospholipid bilayer while PE is strongly destabilizing [30], the high PC/PE ratio in filamentous fungi indicates that their membranes should be more ordered than the yeast ones. On the other hand, sterols have been considered as membrane reinforces because they induce a molecular order of membranes [42,43] and yeast membranes contain slightly (22%) more ergosterol compared to *Trichoderma*.

The stability and fluidity of the phospholipid bilayer is affected by other membranes components, including dolichols. DSC is a useful technique for studying perturbations induced in membranes by diverse compounds. Homogenous phospholipid bilayers exhibit a defined thermal behavior characterized by their calorimetric profile and phase transition temperature, which can be strongly modified when the composition of the bilayers is altered. The changes of these parameters reflect the changes in membrane structure and properties. Our DSC studies showed that the effect of dolichols on the phase behavior of model membranes depends not only on the dolichol structure but also on the phospholipid class. The latter is in line with the results published by [44]. In particular, we found that yeast-type dolichols were unable to perturb PC bilayers but destabilized PE membranes, which has also been reported by [45]. On the other hand, dolichols from *Trichoderma* significantly altered the thermotropic behavior of both PC and PE membranes as gauged by their phase transition enthalpy and cooperativity. However, the temperature of the gel-to-liquid-crystalline phase transition was affected by TrDols only slightly in the case of PE and not at all for PC. These results aid in answering the question of whether the poly-saturated nature of the dolichols from *T. reesei* is of any importance for membrane stability. It is commonly accepted that the rigidity of a molecule is important for its ability to modify the physical properties of membranes. Thus, comparative studies of β-carotene and its fully saturated derivative, perhydro-β-carotene, show that due to the absence of the conjugated double bond system, and therefore higher flexibility of the latter molecule, it has lost the potential to perturb the membrane structure [46]. Flexible molecules apparently can adopt such conformations in the membrane which do not disturb the regular arrangement of the lipid bilayer molecules, thus their effect on the membrane properties is negligible. However, this is likely to be true only up to a certain dose of the “intruder” molecules. One should also note that the saturated bonds in TrDols are most likely located at the ω-end while in the remaining part of the Dol chain, except for the α-residue, the typical polyprenoid arrangement of double bonds remains intact. Such distribution produces different flexibility of the two segments of TrDol molecules.

To further study the effects of dolichols on lipid bilayer fluidity we determined the packing and ordering of the phospholipid bilayers. Most important was a considerable fluidization of both types of model membranes (PC and PE) below their respective transition temperatures, i.e., when they were in the gel phase, by both types of dolichols. This effect was slightly stronger for TrDols compared to ScDols at low doses and markedly stronger for ScDols at higher doses, indicating a rather complex dependence on dolichol structure.

It is worth repeating here that membrane fluidity profoundly affects the functioning of membrane-associated biomolecules, including enzymes. Thus, dolichols of different structure can have considerably different modulatory action on diverse membrane-dependent processes.

## 4. Materials and Methods

### 4.1. Materials

For the isolation and analysis of dolichols, HPLC or p.a. grade organic solvents from POCh were used (Gliwice, Poland), chromatographic columns and TLC plates were from Merck (Darmstadt, Germany), and other chemicals were of p.a. quality and were purchased from Sigma-Aldrich Chemie GmbH (Steinheim, Germany).

For determination of phospholipids, the following standards from Avanti Polar Lipids (Alabaster, AL, USA) were used: 1,2-dimyristoyl-*sn*-glycero-3-phospho-rac-(1-glycerol) sodium salt (14:0/14:0 phosphatidylglycerol (PG)); 1,2-dilauroyl-*sn*-glycero-3-phosphoethanolamine (12:0/12:0 phosphatidylethanolamine (PE)); 1,2-dimyristoyl-*sn*-glycero-3-phosphocholine (14:0/14:0 phosphatidylcholine (PC)); 1,2-dipalmitoyl-*sn*-glycero-3-phospho-(1′-*myo*-inositol) ammonium salt (16:0 phosphatidylinositol (PI)); 1,2-dimyristoyl-*sn*-glycero-3-phospho-l-serine sodium salt (14:0/14:0 phosphatidylserine (PS)), 1′,3′-*bis*[1–dimyristoyl-*sn*-glycero-3-phospho]-*sn*-glycerol ammonium salt (14:0 cardiolipin) and 1,2-dimyristoyl-*sn*-glycero-3-phosphate sodium salt (14:0/14:0 phosphatidic acid (PA)). Standards of polyisoprenoids were from the Collection of Polyprenols, Institute of Biochemistry and Biophysics, PAS, (IBB PAS) Warsaw. Butylated hydroxytoluene (BHT) was from Sigma-Aldrich. All other chemicals were from Avantor Performance Materials (Gliwice, Poland).

### 4.2. Strains and Growth Conditions

*Trichoderma reesei* QM9414, *Aspergillus niger* and *Neurospora crassa* were cultivated in PDB medium (Potato Dextrose Broth), and *Saccharomyces cerevisiae* wild-type strain and *dpm1dr∆/dpm1,3Tr* mutant were cultivated in YPD (1% yeast extract, 1% Bacto-peptone, 2% glucose). The *S. cerevisiae dpm1dr∆/dpm1,3Tr*, carries *dpm1Tr* gene from *Trichoderma* cloned into the p415 GPD LEU plasmid under the *GPD* (glyceraldehyde-3-phosphate dehydrogenase) promoter and the *CYC1* (cytochrome-c-oxidase) terminator [27,47]. The *dpm3Tr* gene was cloned into the pESC-URA vector (Stratagene) under the *GAL10* (UDP glucose 4-epimerase) promoter in cloning sites 2.

All strains were cultivated at 30 °C on a rotary shaker (250 r.p.m.) in 2 L shake flasks containing 1 L of appropriate medium.

### 4.3. Extraction and Purification of Polyisoprenoids

The mycelium was harvested by filtration, suspended in 60% KOH with 0.25% pyrogallol and hydrolyzed at 100 °C for 1 h. Lipophylic products were extracted with diethyl ether and evaporated to dryness, then dissolved in hexane and applied onto a silica gel column equilibrated with hexane. The column was washed with 3% diethyl ether in hexane. The polyisoprenoid fraction was eluted with 10% and 20% hexane in diethyl ether, the two eluates were pooled, evaporated to dryness, dissolved in isopropanol and subjected to HPLC/UV analysis as described earlier [48], with modifications. Briefly, a linear gradient of methanol: water (9:1, *v*/*v*) to methanol: isopropanol: hexane (2:1:1, *v*/*v*/v) was used for elution. Fractions containing polyisoprenoids were collected (based on comparison of their retention times with those of yeast dolichols used as external standards) and their structure was analyzed (see below). For quantitative analysis Dol_13_ was added to the mycelium as an internal standard before hydrolysis. Polyprenol and dolichol standards were from the Collection of Polyprenols (IBB PAS).

### 4.4. Structural Analysis of Dolichols Isolated from S. cerevisiae and Filamentous Fungi

Polyisoprenoids were isolated from fungal cells as described above and subjected to structural analysis with the aid of LC-ESI-QTOF-MS, as described earlier [49].

Briefly, the analysis was performed on an ACQUITY UPLC I-Class Ultra-Performance Liquid Chromatograph (Waters) coupled with the MALDISynapt G2-S HDMS mass spectrometer (Waters) equipped with an electrospray ion source and quadrupole time of flight-type mass analyzer.

The polyisoprenoids were injected in a mixture of methanol: chloroform (1:1, *v*/*v*). The liquid chromatography-MS analysis was carried out at a flow rate of 0.4 mL min^−1^.

### 4.5. Phosphorylation of Dolichols Isolated from T. reesei and S. cerevisiae

Dolichols isolated from *T. reesei* and *S. cerevisiae* were phosphorylated using a standard method [50]. Purified dolichyl phosphates were dissolved in a mixture of chloroform: methanol (2:1, *v*/*v*) and stored at −20 °C until use.

### 4.6. Membrane Preparation

Mycelium was harvested by filtration, washed with water and suspended in 50 mM Tris-HCl, pH 7.4, containing 15 mM MgCl_2_ and 9 mM β-mercaptoethanol. *S.cerevisiae* was cultured in YPD medium to OD_600_ = 1, harvested by centrifugation and resuspended in the above buffer.

Cells were homogenized in a beadbeater with 0.5 mm glass beads and the homogenate was centrifuged at 5000× *g* for 10 min to remove cell debris and unbroken cells. The supernatant was then centrifuged at 100,000× *g* for 1 h. The membrane pellet was homogenized in 50 mM Tris-HCl, pH 7.4, containing 3.5 mM MgCl_2_ and 6 mM β-mercaptoethanol, and used as the source of enzymes. The whole procedure was performed at 4 °C [51].

### 4.7. Protein Assay

Protein concentration was determined in alkaline cooper solution using Folin Phenol reagent according to Lowry et al. [52].

### 4.8. Dolichyl Phosphate Mannose Synthase Activity

DPMS activity was assayed in the membrane fraction by incubation for 5 min at 30°C of 100 µg of membrane protein in a total volume of 50 µL containing 8 × 10^4^ cpm GDP[^14^C]mannose (sp. act. 55 Ci/mol, American Radiolabeled Chemicals, Inc., Saint Louis, MO, USA) and different quantities of dolichyl phosphate (Dol-P) in 40 mM Tris/HCl buffer, pH 7.4 with, 10 mM MgCl_2_ and 0.1% Nonidet P-40 [25,53]. The reaction was stopped by addition of 4 mL of chloroform: methanol (3:2, *v*/*v*). Radioactive dolichyl phosphate mannose was measured in the organic phase in a scintillation counter.

### 4.9. Phylogenetic Tree and 3D Model of DPMS

For phylogeny reconstruction, Dpm1, Dpm2 and Dpm3 sequences from representative fungal genomes were collected from FungiDB [54]. Sequences were aligned using Mafft [55] and phylogenetic tree was built using PhyML [56]. Distant homology searches were performed using Meta-BASIC [57]. 3D models were built using Modeller [58] with manually curated sequence-to-structure alignment taking into account the conservation of functionally essential residues and structural features. Structure visualization was carried out with Pymol (pymol.org).

### 4.10. Determination of Phospholipids

Fungal phospholipids were extracted according to the method of Folch et al. [59], with some modifications. Briefly, fungal biomass was separated from medium by centrifugation (5000× *g*) and disintegrated with 5 mL of methanol: chloroform (1:2, *v*/*v*), 0.05 % BHT and 0.1 mm glass beads on a Mixer Mill MM400 (Retsch, Haan, Germany). The homogenate was supplemented with 5 mL of the above solvent, vortexed for 4 min and centrifuged (6,000× *g*, 2 min). The supernatant was transferred to another Falcon tube. To facilitate the separation of two layers, supernatant was mixed with 2 mL 0.9% NaCl and centrifuged. The lower layer was collected and evaporated under reduced pressure.

The obtained phospholipids were dissolved in 1 mL of methanol: chloroform (4:1, *v*/*v*). The polar lipids were separated and identified using an Agilent 1200 HPLC system (Santa Clara, CA, USA) and a 4500 Q-TRAP mass spectrometer (Sciex, Framingham, MA, USA) equipped with an ESI source. For this, 10 μL of phospholipid extract was injected onto a Kinetex C8 column (150 mm × 2.1 mm, particle size: 5 μm; Phenomenex, Torrance, CA, USA) heated at 40 °C, with the flow rate of 500 µL·min^−1^. Water (A) and methanol (B) were applied as a mobile phase, both containing 5 mM ammonium formate. The solvent profile was 70% B for 15 s, increased to 95% B over 1 min and maintained at 95% B for 17 min, then returned to 70% B over 3 min. The following MS settings were applied: spray voltage—4500 V, curtain gas 25, nebulizer gas 60, auxiliary gas 50, and ion source temperature 600 °C. The data analysis was conducted with Analyst™ v1.6.2 software (Sciex, Framingham, MA, USA).

The phospholipids were identified according to the methods described earlier [29]. Then, using a phospholipid standard for each phospholipid class the quantity of a given phospholipid species from each class was calculated.

### 4.11. Determination of Ergosterol

Ergosterol was extracted according to Bernat et al [29] with some modifications. Fungal biomass was lyophilized and homogenized in methanol: chloroform 2:1, *v*/*v*) using a ball mill (FastPrep-24, MP-Biomedicals, Santa Ana, CA, USA). Organic phase was washed with water, evaporated and dissolved in methanol: chloroform (4:1, *v*/*v*). Ergosterol was measured using an Agilent 1200 HPLC system as above for phospholipids determination.

### 4.12. Differential Scanning Calorimetry (DSC) Analysis

Appropriate amounts of phospholipid in chloroform (HPLC grade) and polyprenols dissolved in n-hexane were mixed in a glass test tube. The organic solvent was gently evaporated to dryness under a stream of nitrogen until a thin film was formed on the test tube wall. The film was hydrated with distilled water and multilamellar liposomes (MLV) were formed by vortexing the sample for 10 min at a temperature above the main phase transition temperature (T_m_) of pure phospholipid, i.e., for 1,2-dipalmitoyl-*sn*-glycero-3-phosphatidylcholine (16:0/16:0 phosphatidylcholine (DPPC)) the temperature was 50 °C, and for 1,2-dimyristoyl-*sn*-glycero-3-phosphatidylethanolamine (14:0/14:0 phosphatidylethanolamine (DMPE)) 70 °C, and sonicated. The final DPPC and DMPE concentration in the MLV was 1 mM and the polyprenol content ranged from 1 to 50 mol%. Differential scanning colorimetry of the MLV was performed using a NANO DSC Series III System with a platinum capillary cell (TA Instruments, New Castle, DE, USA) with an active volume of 300 μL. To avoid bubble formation, the samples were degassed at 30.4–50.7 kPa for 15 min then placed in the sample cell; distilled water was used as a reference. The cells were sealed and thermally equilibrated for 10 min at the starting temperature. Sample pressure was 0.3 MPa. Heating/cooling rates were 1 °C·min^−1^ and the scans were recorded in the temperature range of 20–60 °C for DPPC and 30–70 °C for DMPE. The accuracy of temperature determination was ±0.1 °C. Heating scans were carried out first. The reference scan was subtracted from sample scans. Transition temperatures were calculated using the software package supplied by TA Instruments. The measurements were performed in triplicate.

### 4.13. Fluorescence Anisotropy of DPH

The hydrophobic fluorescent probe l,6-diphenyl-l,3,5-hexatriene (DPH) (Sigma-Aldrich) was incorporated into small unilamellar vesicles (SUVs) formed from DPPC or egg yolk lecithin (EYPC), type V-E, containing various proportions of dolichols. The SUVs were prepared according to the injection method [60]. Briefly, solutions of phospholipid, polyprenol and DPH were mixed and the solvent was evaporated under nitrogen. The dry residue was dissolved in a small volume of ethanol and slowly injected into water with continuous stirring at a temperature above that of the main phase transition of the lipid. The final phospholipid concentration was 0.5 mM, the phospholipid/DPH ratio 1:1000, and the polyprenol content varied between 1 and 30 mol%. The experiments were carried out at 20 °C for EYPC and at 20 °C and 50 °C for DPPC. Fluorescence anisotropy measurements were performed using a FluoroMax-P spectrofluorometer (Horiba Scientific, Besheim, Germany) equipped with a thermostated cuvette holder and stirrer. Excitation and emission were at 360 nm and 450 nm, respectively. The anisotropy value (r) was calculated from the equation:

$$r=\frac{I_{VV}-I_{VH}}{I_{VV}+2GI_{VH}}$$

where *I_VV_* and *I_VH_* are the parallel and perpendicular components of DPH emission with respect to the direction of the polarized excitation, and G is the instrument correction factor which compensates for the slightly unequal horizontal and vertical excitation intensities. The measurements were performed in triplicate for independently prepared samples.

## 5. Conclusions

These studies showed that filamentous fungi synthesize atypical poly-saturated dolichols not found in other Eukaryotes. The most saturated dolichols with up to ten additional saturated bonds were found in *T. reesei*.

The activity of dolichol-dependent enzyme, DPMS, was much lower in *Trichoderma* than in *S. cerevisiae*, and the *Trichoderma* dolichols were a much poorer substrate than were yeast dolichols.

3D model studies revealed that DPMS from *Trichoderma* and yeast may differ in the strength of Dol-P binding. The structural differences at the ω-end of dolichols are unlikely to affect the interaction with DPMS catalytic subunit, although an effect on the interactions with the membrane-spanning parts of the enzyme cannot be excluded.

The high PC/PE ratio in membranes from filamentous fungi suggests a higher stability of the membrane bilayer than in yeast. Studies on model membranes showed that the two types of dolichols differ in their membrane-destabilizing effect, which also depended on the membrane composition.

## Figures and Tables

**Table 1:** Dolichols found in filamentous fungi, *Trichoderma reesei* (T) *Aspergillus niger* (A) and *Neurospora crassa* (N).

Dolichol Species	No of Additional Saturated Bounds	Dol-18			Dol-19			Dol-20			Dol-21			Dol-22			Dol-23		
		T	A	N	T	A	N	T	A	N	T	A	N	T	A	N	T	A	N
dolichol	0	+			+	+	+	+	+	+		+	+		+	+		+	
dihydrodolichol	1	+			+	+	+	+	+	+		+	+		+	+		+	
tetrahydrodolichol	2	+	+		+	+		+	+			+			+			+	
hexahydrodolichol	3	+			+			+											
octahydrodolichol	4	+			+			+											
dekcahydrodolichol	5	+			+			+											
dodecahydrodolichol	6	+			+			+											
tetradecahydrodolichol	7	+			+			+											
hexadecahydrodolichol	8	+			+			+											
oktadecahydrodolichol	9				+			+											
icosahydrodolichol	10				+			+											

Dol-18, 19, 20, 21, 22, 23—dolichols containing from 18 to 23 isoprene units, + marks presence of a given compound in fungal species. *Trichoderma* is marked by deep gray -, *Aspergillus* by middle gray and *Neurospora* by light gray color.

**Table 2:** Kinetic parameters of dolichyl phosphate mannose synthase from *S. cerevisiae* and *T. reesei* for different dolichol phosphates.

Dolichols	*S. cerevisiae*		*T. reesei*		*T. reesei* Dpm1,3 Expressed in *S. cerevisiae*	
	Vmax [pmol/mg protein/5min]	Km [ng]	Vmax [pmol/mg protein/5min]	Km [ng]	Vmax [pmol/mg protein/5min]	Km [ng]
Pig liver (Dol-P18/19)	825	2	220	2.5	819	7.3
*S. cerevisiae* (dominating Dol-P16)	1897	25,550	313	48,180	1710	25,550
*T. reesei*	264	46,400	34	34,000	348	43,800
Dol-P11	905	730	298	3500	821	730

Activity of DPMS from *Trichoderma* was analyzed in *T. reesei* and in *S. cerevisiae* mutant (Dpm1, 3) carrying Dpm1and Dpm3 proteins from *Trichoderma*.

**Table 3:** Profile of fatty acids in membranes from yeast and filamentous fungi.

Fatty Acid (Common Name)	Content of Fatty Acid [%]			
	*S. cerevisiae*	*T. reesei*	*A. niger*	*N. crassa*
10:0 Capric	0.66 ± 0.04			
12:0 Lauric	0.99 ± 0.53			
14:0 Miristic	0.50 ± 0.07	0.37 ± 0.04	0.12 ± 0.035	0.12 ± 0.09
15:0 Pentadecylic			0.42 ± 0.026	0.13 ± 0.01
16:0 Palmitic	12.72 ± 0.96	28.87 ± 1.83	15.13 ± 1.48	7.95 ± 0.01
16:1 Palmitoleic	**55.11 ± 3.27**		0.77 ± 0.15	2.06 ± 0.01
17:0 Margaric			0.25 ± 0.05	
18:0 Stearic	3.73 ± 0.26	10.74 ± 0.95	4.34 ± 1.25	2.70 ± 0.15
18:1 Oleic	26.26 ± 1.74	23.36 ± 0.17	18.22 ± 0.1	9.94 ± 0.27
18:2 Linoleic		**34.82 ± 2.86**	**56.22 ± 2.55**	**63.67 ± 0.55**
18:3 Linolenic		1.27 ± 0.11	4.5 ± 0.2	11.59 ± 0.4
20:0 Arachidic		0.19 ± 0.03		0.19 ± 0.01
22:0 Behenic				0.39 ± 0.05
24:0 Lignoceric		0.34 ± 0.01		1.23 ± 0.13

Dominating fatty acid in bold and underlined. Means ± standard deviation of three independent analysis is shown.

**Table 4:** Thermodynamic parameters of fully hydrated multilamellar DPPC liposomes supplemented with *S. cerevisiae* or *T. reesei* dolichols.

Compound		Pretransition Heating			Main Transition Heating			Pretransition Cooling			Main Transition Cooling		
	mol %	∆H [kJ/mol]	∆S [kJ/mol K]	T_p_ [°C]	∆H [kJ/mol]	∆S [kJ/mol·K]	T_m_ [°C]	∆H [kJ/mol]	∆S [kJ/mol·K]	T_p_ [°C]	∆H [kJ/mol]	∆S [kJ/ mol·K]	T_m_ [°C]
DPPC													
		6.72	0.022	36.03	31.69	0.101	42.02	1.59	0.005	29.82	35.57	0.113	41.04
+ *S. cerevisiae* dolichols													
	1	3.99	0.013	36.07	34.97	0.111	42.00	1.30	0.004	29.86	34.97	0.111	41.06
	5	4.26	0.014	36.00	36.23	0.115	42.02	1.97	0.007	29.72	36.22	0.115	41.06
	15	3.51	0.011	35.90	35.41	0.113	42.02	1.31	0.004	29.50	35.41	0.113	41.05
	30	4.07	0.013	35.78	35.49	0.113	42.02	0.88	0.003	29.60	35.49	0.113	41.02
	50	3.58	0.012	35.83	33.91	0.108	42.00	0.76	0.003	29.70	33.91	0.108	40.98
+ *T. reesei* dolichols													
	1	3.81	0.012	36.28		0.100	42.05	1.23	0.004	29.98	31.31	0.100	41.10
	5	2.82	0.005	36.50		0.105	42.17	1.00	0.003	30.91	32.86	0.105	41.11
	15	0.36	0.001	36.62		0.119	42.33	-	-	-	37.31	0.119	41.16
	30	-	-	-		0.116	42.26	-	-	-	36.31	0.116	41.06
	50	-	-	-		0.117	41.93	-	-	-	36.65	0.117	40.97

Changes were determined in heating and cooling modes. The rate of temperature change was 1 °C min^−1^. The accuracy for the main phase transition temperature determination is ± 0.01 °C and of enthalpy change ± 0.8 kJ/mol.

**Table 5:** Thermodynamic parameters of fully hydrated multilamellar 1,2-dimyristoyl-*sn*-glycero-3-phosphatidylethanolamine (DMPE) liposomes supplemented with *S. cerevisiae* or *T. reesei* dolichols.

Compound		Heating			Cooling		
	mol %	∆H [kJ/mol]	∆S [kJ/ mol·K]	T_m_ [°C]	∆H [kJ/mol]	∆S [kJ/mol·K]	T_m_ [°C]
DMPE							
		22.84	0.071	50.67	22.14	0.069	49.26
+ *S. cerevisiae* dolichols							
	1	23.56	0.073	50.18	26.54	0.083	48.56
	5	22.26	0.069	49.94	25.92	0.081	48.26
	15	20.85	0.065	49.92	25.48	0.079	48.21
	30	21.61	0.067	49.92	22.43	0.070	48.17
	50	16.84	0.052	49.87	17.97	0.056	48.14
+ *T. reesei* dolichols							
	1	22.88	0.071	50.49	22.67	0.071	48.66
	5	23.35	0.072	50.33	23.77	0.074	48.76
	15	24.14	0.075	50.57	26.75	0.083	49.00
	30	18.98	0.059	51.09	20.07	0.062	49.41
	50	10.28	0.032	51.03	9.72	0.030	49.39

Changes were determined in heating and cooling modes. The rate of temperature change was 1 °C min^−1^. The accuracy for the main phase transition temperature determination is ± 0.01 °C and of enthalpy change ± 0.8 kJ/mol.

## Supplementary Materials

Supplementary materials can be found at https://www.mdpi.com/1422-0067/20/12/3043/s1.
