# Supplementary material for: Poly-Saturated Dolichols from Filamentous Fungi Modulate Activity of Dolichol-Dependent Glycosyltransferase and Physical Properties of Membranes
Source: Int J Mol Sci. 2019 Jun 21;20(12):3043. doi: 10.3390/ijms20123043 (PMC6628320; doi:10.3390/ijms20123043)
Supplement: Supplementary file 1 [file ijms-20-03043-s001.pdf]

Table 1S

Molecular species of dolichols from *T. reesei* identified by mass spectrometry

Compound	Measured m/z [M+NH4]+	Calculated m/z [M+NH4]+	ΔM (ppm)	Molecular Formula [M+NH4]+
Dol18	1263.1854	1263.1874	-1.6	C90H152NO
Dol18+2H	1265.2054	1265.2030	1.9	C90H154NO
Dol18+4H	1267.2115	1267.2187	-5.7	C90H156NO
Dol18+6H	1269.2347	1269.2343	0.3	C90H158NO
Dol18+8H	1271.2518	1271.2500	1.4	C90H160NO
Dol18+10H	1273.2626	1273.2656	-2.4	C90H162NO
Dol18+12H	1275.2828	1275.2813	1.2	C90H164NO
Dol18+14H	1277.2969	1277.2969	0.0	C90H166NO
Dol18+16H	1279.3203	1279.3126	6.0	C90H168NO
Dol19	1331.2476	1331.2500	-1.8	C95H160NO
Dol19+2H	1333.2733	1333.2656	5.8	C95H162NO
Dol19+4H	1335.2847	1335.2813	2.5	C95H164NO
Dol19+6H	1337.3055	1337.2969	6.4	C95H166NO
Dol19+8H	1339.3120	1339.3126	-0.4	C95H168NO
Dol19+10H	1341.3359	1341.3282	5.7	C95H170NO
Dol19+12H	1343.3453	1343.3439	1.0	C95H172NO
Dol19+14H	1345.3644	1345.3595	3.6	C95H174NO
Dol19+16H	1347.3768	1347.3752	1.2	C95H176NO
Dol19+18H	1349.3907	1349.3908	-0.1	C95H178NO
Dol19+20H	1351.4063	1351.4065	-0.1	C95H180NO
Dol20	1399.3210	1399.3126	6.0	C100H168NO
Dol20+2H	1401.3325	1401.3282	3.1	C100H170NO
Dol20+4H	1403.3456	1403.3439	1.2	C100H172NO
Dol20+6H	1405.3599	1405.3595	0.3	C100H174NO
Dol20+8H	1407.3757	1407.3752	0.4	C100H176NO
Dol20+10H	1409.3931	1409.3908	1.6	C100H178NO
Dol20+12H	1411.4036	1411.4065	-2.1	C100H180NO
Dol20+14H	1413.4319	1413.4221	6.9	C100H182NO
Dol20+16H	1415.4371	1415.4378	-0.5	C100H184NO
Dol20+18H	1417.4601	1417.4534	4.7	C100H186NO
Dol20+20H	1419.4846	1419.4691	10.9	C100H188NO

Analysis was performed by LC-ESI-QTOF-MS [47]

Figure 1S

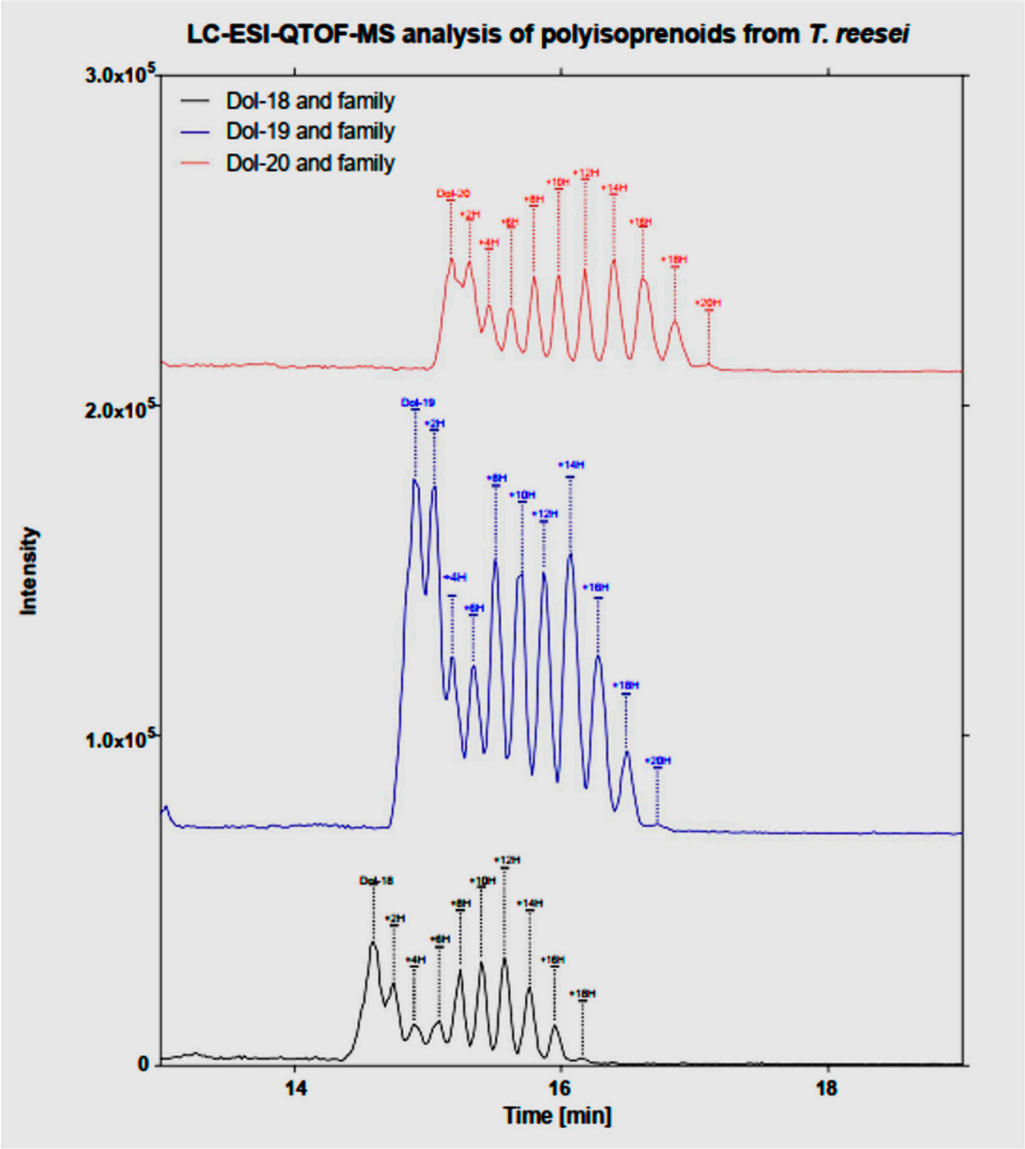

Table 2S

Continuous lines between points represent theoretical fits to the experimental data by applying equation: y = Intercept + B1*x^1 + B2*x^2. Parameters characterizing dependence of DPH fluorescence anisotropy on dolichol content obtained from the fitting are given in Table.

	Intercept		B1		B2		Statistics
	Value	Standard Error	Value	Standard Error	Value	Standard Error	Adj. R-Square
TrDols_EYPC	0.21841	0.0013	-0.00283	3.45726E-4	3.45926E-5	1.22683E-5	0.98629
TrDols DPPC 20	0.2161	0.00544	-0.00312	0.00166	7.7547E-5	6.12485E-5	0.97785

TrDols DPPC 50	0.05437	8.69298E-4	6.73167E-4	2.76566E-4	1.53187E-6	8.89231E-6	0.96685
ScDols_EYPC	0.21841	0.0013	-0.00283	3.45726E-4	3.45926E-5	1.22683E-5	0.98629
ScDols 20	0.21841	0.0013	-0.00283	3.45726E-4	3.45926E-5	1.22683E-5	0.98629
ScDols DPPC 50	0.05613	4.29571E-4	5.68912E-4	9.90187E-5	-1.93783E-5	3.40209E-6	0.88717
